# Structural analysis of inhibition of *E. coli *methionine aminopeptidase: implication of loop adaptability in selective inhibition of bacterial enzymes

**DOI:** 10.1186/1472-6807-7-84

**Published:** 2007-12-19

**Authors:** Ze-Qiang Ma, Sheng-Xue Xie, Qing-Qing Huang, Fa-Jun Nan, Thomas D Hurley, Qi-Zhuang Ye

**Affiliations:** 1 High Throughput Screening Laboratory, University of Kansas, Lawrence, Kansas 66047, USA; 2 Chinese National Center for Drug Screening, Shanghai Institute of Materia Medica, Chinese Academy of Sciences, Shanghai 201203, China; 3 Department of Biochemistry and Molecular Biology, Indiana University School of Medicine, Indianapolis, IN 46202, USA

## Abstract

**Background:**

Methionine aminopeptidase is a potential target of future antibacterial and anticancer drugs. Structural analysis of complexes of the enzyme with its inhibitors provides valuable information for structure-based drug design efforts.

**Results:**

Five new X-ray structures of such enzyme-inhibitor complexes were obtained. Analysis of these and other three similar structures reveals the adaptability of a surface-exposed loop bearing Y62, H63, G64 and Y65 (the YHGY loop) that is an integral part of the substrate and inhibitor binding pocket. This adaptability is important for accommodating inhibitors with variations in size. When compared with the human isozymes, this loop either becomes buried in the human type I enzyme due to an N-terminal extension that covers its position or is replaced by a unique insert in the human type II enzyme.

**Conclusion:**

The adaptability of the YHGY loop in *E. coli *methionine aminopeptidase, and likely in other bacterial methionine aminopeptidases, enables the enzyme active pocket to accommodate inhibitors of differing size. The differences in this adaptable loop between the bacterial and human methionine aminopeptidases is a structural feature that can be exploited to design inhibitors of bacterial methionine aminopeptidases as therapeutic agents with minimal inhibition of the corresponding human enzymes.

## Background

Methionine aminopeptidase (MetAP) removes the N-terminal methionine residue from nascent proteins in all types of cells [1]. Prokaryotic cells express only one MetAP, and its essentiality was demonstrated by the lethality of its deletion from *Escherichia coli *[2] and *Salmonella typhimurium *[3]. MetAP is therefore a potential target for developing novel broad spectrum antibacterial drugs [4]. Eukaryotic cells have two types of MetAP (type I and type II), and deletion of both MetAP genes in *Saccharomyces cerevisiae *was shown to be lethal [5,6]. Fumagillin and its analogues TNP-470 and ovalicin are potent antiangiogenic compounds and are also selective inhibitors of human type II MetAP [7-9]. The antiproliferative bengamides inhibit both types of human MetAP [10]. Therefore, human MetAPs may also serve as targets for development of new anticancer therapeutics.

Early MetAP inhibitors were derived from peptide substrates or the cleavage product methionine, such as the peptic inhibitor (3R)-amino-(2S)-hydroxyheptanoyl-L-Ala-L-Leu-L-Val-L-Phe-OMe (*K*_i _5 μM) [11] and norleucine phosphonate (NleP) [12]. Both are considered as transition state inhibitors. Although these compounds are not desired as therapeutic agents, structural studies of their complexes with MetAP have provided valuable insight of the catalysis and inhibition of MetAP [12-14]. Fumagillin, a natural product, and its analogues are a unique class of MetAP inhibitors that covalently modify a conserved histidine residue at the active site (H79 of *E. coli *MetAP, and the equivalent H231 of human type II MetAP) [9,15,16]. Several classes of non-peptidic and reversible MetAP inhibitors have been identified recently, such as furancarboxylic acids [17,18], thiabendazole and other thiazole-containing compounds [17,19-21], triazole-based derivatives [22-24], and sulfonamides [25,26]. However, structural analysis of these nonpeptidic inhibitors in complex with MetAP showed that inhibition by many of the thiazole and triazole-containing compounds and sulfonamides is metal-mediated, and they bind to the active site of enzyme through a divalent metal ion with one of the conserved active site histidines (most with H97, and some with H181; both are *E. coli *MetAP numbering) [19,21,25]. It has been pointed out that formation of such complexes may be an artefact during crystallization or in in vitro assays using high metal concentrations [14,19,27], and whether there are enough free metal ions available inside cells to form such inhibitor-enzyme complexes is a question.

MetAP was initially characterized as a Co(II) enzyme because of reproducible activation of the apoenzyme by Co(II) [5,28]. Many X-ray structures of MetAPs with or without a ligand bound [29] show a dinuclear metal site inside the active site pocket that has five conserved residues D97, D108, H171, E204 and E235 (*E. coli *MetAP numbering) as metal ligands and filled with two Co(II) ions. The metal ion used to form the inhibitor-enzyme complexes mentioned above is neither of the metal ions, but an additional one close to the dinuclear site. In addition to Co(II), other divalent metals such as Mn(II), Ni(II), Zn(II), and Fe(II) have been shown to activate the enzyme in vitro as well [30,31]. It is not known which of the metal ions is actually used by MetAP under physiological conditions, but speculation favors Fe(II), Zn(II) or Mn(II) for this role [23,31,32].

**Figure 1:**
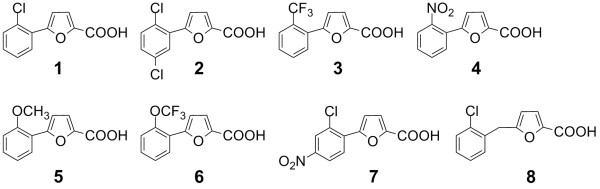
**Chemical structures of the inhibitors used in this study**. X-ray structures of *E. coli *MetAP complexed with 1–3 have been reported previously [17, 33], and those with 4–8 are reported here. They are potent inhibitors of the Mn(II)-form of *E. coli *MetAP with IC50 values of 0.51, 0.69, 0.29, 1.1, 0.56, 0.37, 1.6 and 1.2 μM, respectively [18].

By high throughput screening of a diverse chemical library of small organic compounds, we have discovered furancarboxylic acids as MetAP inhibitors with high selectivity for the Mn(II)-form of the enzyme [17]. Importantly, they remain potent as MetAP inhibitors at low and physiologically more relevant metal concentration [27]. X-ray structures showed that they directly interact with the two metals at the dinuclear site without requiring the additional metal for binding [17,33]. Several derivatives of the screening hits were synthesized to study structure-function relationships for their inhibitory potency and metalloform-selectivity [18]. To elucidate the chemical basis for metalloform-selective MetAP inhibition in greater detail, we have now carried out structural studies of *E. coli *MetAP in complex with these metalloform-selective inhibitors. We report here five new X-ray crystal structures of the Mn(II)-form of *E. coli *MetAP, each complexed with a different inhibitor (Fig. 1). Analysis of these and other complexes identifies an adaptable loop of the active site pocket as an important structural feature of the enzyme that may be exploited to achieve selective inhibition of bacterial MetAP enzymes vs. mammalian counterparts.

## Results and Discussion

### Overall structure of *E. coli *MetAP in complex with the inhibitors

**Figure 2:**
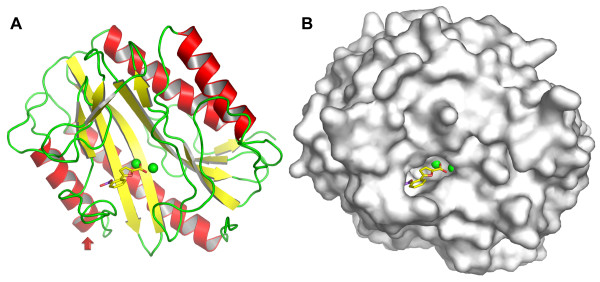
**Overall structure of *E. coli *MetAP complexed with an inhibitor**. Ribbon (A) and surface (B) drawings are shown for one of the structures with inhibitor **4 **situated at the active site. The two Mn(II) ions are shown as green spheres, and the inhibitor is shown as sticks (yellow, carbon; red, oxygen; and blue, nitrogen). In ribbon drawing, the secondary structures are coded as red for α-helices, yellow for β-sheets, and green for loops and other structures. The YHGY loop (Y62, H63, G64 and Y65) is indicated by a red arrow.

The crystals generated by hanging-drop method consistently produced high-quality diffraction data for structural solution to resolution from 1.6 to 1.9 Å (Table 1). All eight structures, including the five new ones, have the typical "pita-bread" fold (Fig. 2A) found in other MetAP structures [29], and each of them contains two Mn(II) ions at the dinuclear metal site. A single molecule of inhibitor sits in the shallow active site pocket (Fig. 2B). A surface-exposed loop containing Y62, H63, G64 and Y65 (the YHGY loop) is an integral part of the active site pocket. Notably, this loop adapts different positions in these structures to accommodate different inhibitors, and the implications of this adaptability for inhibitor design will be discussed further below.

**Table 1:** X-ray data collection and refinement statistics

Inhibitor	**4**	**5**	**6**	**7**	**8**
Inhibitor code	B23	B21	A04	A05	B18
PDB code	2Q92	2Q93	2Q94	2Q95	2Q96
*Cell Parameters*					
Space group	*P*2_1_	*P*2_1_	*P*2_1_	*P*2_1_	*P*2_1_
a (Å)	38.1	39.1	38.2	38.8	39.3
b (Å)	61.0	62.3	60.6	62.4	62.0
c (Å)	50.7	52.4	50.6	52.4	52.4
β (deg)	104.9	108.8	104.8	108.4	108.8
*X-ray Data Collection*					
Resolution range (Å)					
Overall	30-1.9	20-1.6	20-1.6	24-1.7	20-1.6
Outer shell	2.0-1.9	1.7-1.6	1.7-1.6	1.8-1.7	1.7-1.6
Collected reflections	63,846	108,452	100,161	93,908	112,816
Unique reflections	17,799	29,294	27,719	26,219	31,502
Completeness (%) ^a^	100 (100)	93 (89)	99 (98)	100 (100)	100 (100)
I/σ (I)^a^	18.1 (4.7)	23.6 (11.1)	20.8 (2.5)	17.3 (2.8)	23.5 (7.2)
*R*_merge _(%) ^a^	5.4 (22.1)	3.0 (9.9)	4.4 (36.3)	5.5 (38.9)	3.8 (13.8)
*Refinement Statistics*					
*R *(%)	20.3	22.6	21.3	21.4	21.1
*R*_free _(%)	23.8	25.1	23.8	23.9	23.5
RMSD bonds (Å)	0.006	0.005	0.005	0.005	0.004
RMSD angles (deg)	1.32	1.32	1.32	1.33	1.30
No. of solvent molecules	133	224	194	195	234
<B> enzyme (Å ^2^)	21.4	11.3	20.9	19.0	11.8
<B> inhibitor (Å ^2^)	25.8	13.3	18.4	16.3	12.1
<B> water (Å ^2^)	27.0	18.6	28.9	25.8	18.8

^a ^Numbers given in parentheses corresponding to the outer shell of data.

### Binding of the inhibitors to *E. coli *MetAP

**Figure 3:**
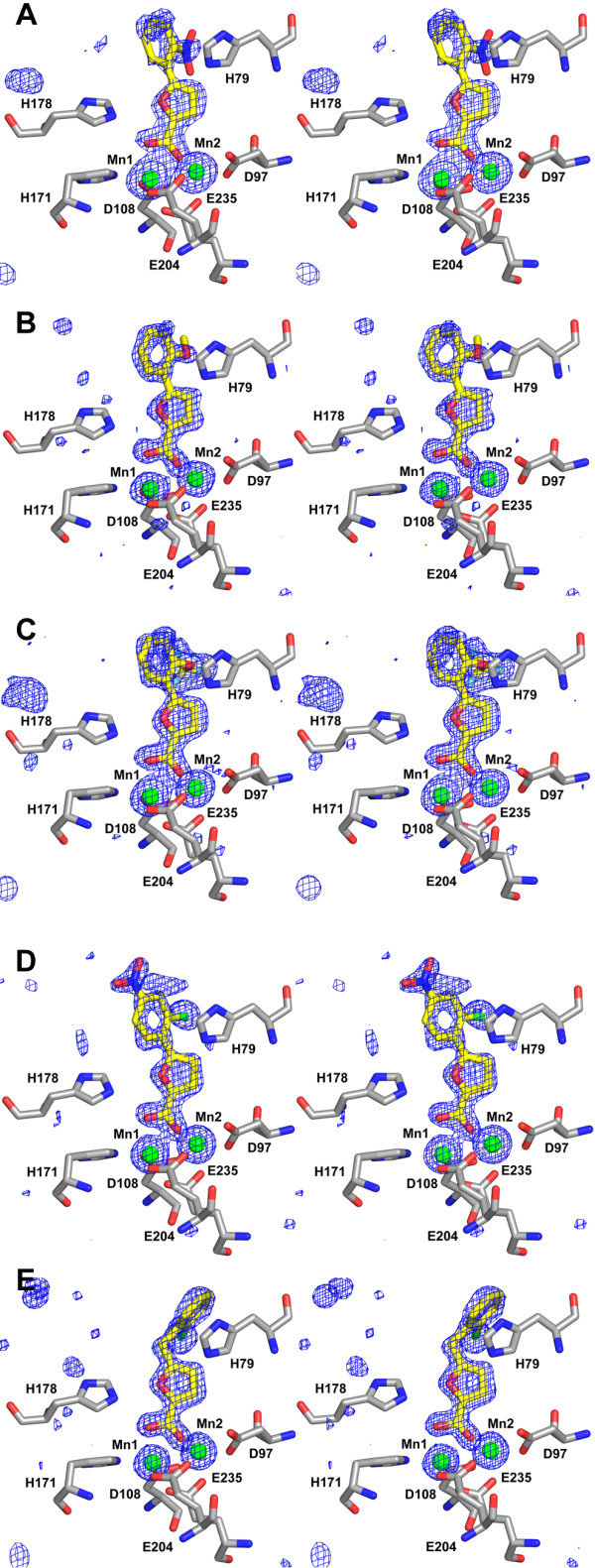
**Binding modes of the inhibitors at the active site of *E. coli *MetAP**. In the stereo views, only five conserved residues that coordinate with Mn(II) ions (D97, D108, H171, E204, E235) and two conserved histdines (H79, H178) are shown. The bound inhibitors are **4 **(A), **5 **(B), **6 **(C), **7 **(D), and **8 **(E), respectively. The colour scheme is as follows: gray, carbon (protein residues); yellow, carbon (inhibitor); blue, nitrogen; red, oxygen; green, chlorine; and cyan, fluorine. Mn(II) ions are shown as green spheres. SigmaA-weighted *F*_obs_-*F*_calc _standard omit maps (inhibitor and metal ions were not included in the model for the structure-factor calculation) are shown superimposed on the refined structures as blue meshes contoured at 3.5 standard deviations of the resulting electron density map.

Common features of the Mn(II)-form selective inhibitors **4**–**8 **bound to *E. coli *MetAP in the five new structures are that all use their carboxylate group to coordinate with the two Mn(II) ions at the dinuclear metal site and all take a non-coplanar or twisted conformation for the two aromatic rings (Fig. 3), consistent with our previously reported structures of *E. coli *MetAP complexed with **1**–**3 **[17]. The twisted conformation found in all of the Mn(II)-form-selective inhibitors **1**–**8 **is in agreement with the requirement of a hydrophobic ortho-substitution, such as chlorine, on the phenyl ring for inhibitory activity [17,18]. This twisting is usually explained in terms of repulsion between ortho hydrogens or substituents in a planar conformation. The twist angles observed in the MetAP complexes of **1**–**8 **range from the smallest 23.3° for **6 **to the largest 52.9° for **4**, suggesting that in general, the phenylfuran-based inhibitors dock into the active site in a conformation that may correspond to a minimum-energy solution conformation. This in turn would enhance their binding by decreasing the fraction of binding energy that would be "wasted" to distort the molecule to a less-favorable conformation in the bound state.

### Adaptability of the YHGY loop in the inhibitor binding pocket

**Figure 4:**
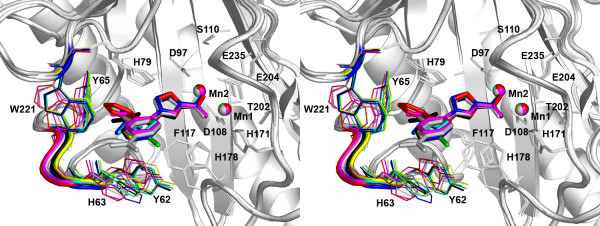
**Adaptability of residues to the bound inhibitors at the active site**. In this stereo view, all eight structures, each with an inhibitor (**1**–**8**) bound, were superimposed. The inhibitors are shown as thick sticks, and the nearby protein residues are shown as thin sticks. The two Mn(II) ions are shown as spheres and labelled as Mn1 and Mn2. The YHGY loop and residues Y62, H63, Y65 and W221, as well as Mn(II) ions, of each structure are coloured the same as the corresponding inhibitor: **1**, yellow; **2**, green; **3**, cyan; **4**, black; **5**, magenta; **6**, blue; **7**, grey; and **8**, red.

With the eight structures with similar inhibitors **1**–**8 **available, we aligned these structures to identify similarities, as well as differences, among them. All structures aligned well with root mean square deviations (rmsd) ranging from 0.112 to 0.356 Å for the Cα carbons of residues 4–256 (Table 2). With this level of overall similarity, it is very noticeable that some residues are essentially immobile while others occupy distinctly different positions when different inhibitors are bound (Fig. 4). The residues forming the dinuclear metal site (H171, D108, E204, E235 and D97), as well as the nearby residues S110, T202, F177, H178 and H79, show little change in position upon binding of any of these inhibitors. In contrast, residues Y62, H63, G64, and Y65 in the YHGY loop, as well as residue W221, moved significantly. The largest changes occur between complexes containing inhibitors **1 **and **8**, where the respective Cα carbons of residues Y62, H63, G64 and Y65 differ in positions by 1.00, 1.58, 1.62 and 1.36 Å, respectively. The rmsd value for all Cα carbons of residues 4–256 is also the largest at 0.356 Å between the two structures. Clearly, the loop is pushed outwards to accommodate the extra volume occupied by inhibitor **8**, and the surface exposure of the loop allows it to more readily accommodate the inhibitor. The same is true for other inhibitors, and the loop show a great adaptability. This flexibility allows the binding pocket to adapt to different shapes of the bound inhibitors. In principle, there could be an energetic penalty to pay (in terms of weaker binding) for moving this loop. However, the fact that inhibitors **1**–**8 **are roughly equipotent [18] suggests that any energetic penalty for moving the loop may be compensated by other favorable interactions, such as compensating desolvation effects.

**Table 2:** rmsd values generated by pair-wise comparisons of the eight complex structures^a^

	2EVM	2EVC	2Q92	2Q93	2Q94	2Q95	2Q96
1XNZ	0.161 (0.006)	0.140 (0.005)	0.231 (0.007)	0.333 (0.036)	0.254 (0.020)	0.316 (0.028)	0.356 (0.051)
2EVM		0.128 (0.004)	0.175 (0.001)	0.292 (0.026)	0.185 (0.008)	0.283 (0.018)	0.309 (0.039)
2EVC			0.192 (0.004)	0.307 (0.029)	0.200 (0.015)	0.289 (0.020)	0.319 (0.043)
2Q92				0.230 (0.022)	0.122 (0.009)	0.216 (0.014)	0.252 (0.035)
2Q93					0.245 (0.017)	0.112 (0.002)	0.117 (0.005)
2Q94						0.240 (0.012)	0.241 (0.028)
2Q95							0.170 (0.009)

^a ^The pdb codes and the bound inhibitors in the eight structures are: 1XNZ, **1**; 2EVM, **2**, 2EVC, **3**; 2Q92, **4**; 2Q93, **5**; 2Q94, **6**; 2Q95, **7**; and 2Q96, **8**. These structures were aligned in a pair-wise fashion using the Cα carbons of residues 4–256; the resulting rmsd values (in Å) are presented. Numbers given in parentheses are reductions of rmsd (in Å) when the same calculations were carried out with exclusion of residues 61–64 (the YHGY loop).

### Statistical analysis of the loop adaptability

**Figure 5:**
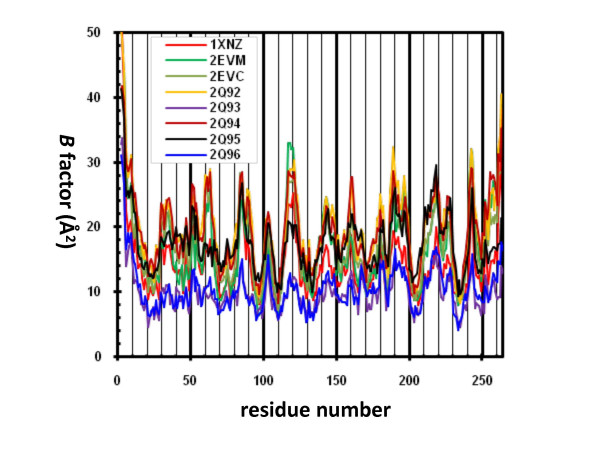
**Distribution of B factors over the residues of the complex structures**. Only B-factors for Cα carbons are shown.

The YHGY loop (Y62, H63, G64, and Y65) forms part of the substrate and inhibitor binding pocket and has direct contact with the bound inhibitors. Positional uncertainty of these atoms in the structures is reflected in their B-factors. The B-factor values of atoms in this loop refined to high values in some of the structures (2EVM, 2EVC, 2Q92, 2Q94), indicating that these atoms are not as well ordered in those structures as in others (Fig. 5). However, the same atoms in some of the structures (2Q93, 2Q96) have low B-factors. The atoms in the YHGY loop do not consistently show high value for B-factors in the presence of a bound inhibitor, in comparison with other parts of the molecule. One of the reasons for this observation is that the value of B-factor is affected by interactions of the atom with the bound inhibitor. Therefore, the absolute value of their B-factors is not a good indicator of their adaptability.

Although the eight structures show good overall alignment as indicated by their small rmsd values during pair-wise comparisons (Table 2), the YHGY loop assumes different positions when different inhibitors are present (Fig. 4). It is revealing that when the residues 61–64 (the YHGY loop) are excluded from the rmsd calculation, the values dropped significantly for some of the pairs (Table 2), indicating a major contribution of the YHGY loop to the rmsd values for these pairs. Notably, the rmsd value between 1XNZ (complex with **1**) and 2Q96 (complex with **8**) showed the biggest drop from 0.356 Å to 0.304 Å with a reduction of 0.051 Å (14%) just by removing four out of 253 residues for the rmsd calculation.

**Figure 6:**
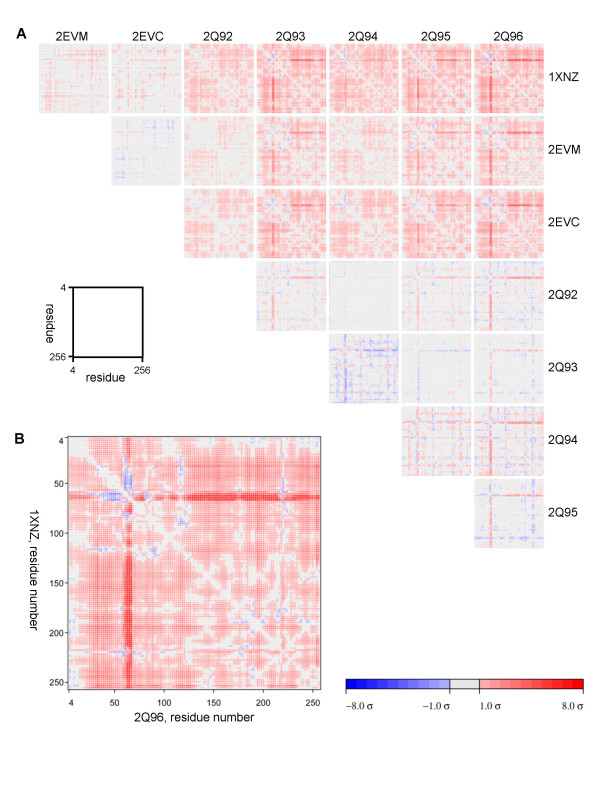
**Error-scaled difference distance matrices generated by pair-wise comparisons of the eight complex structures**. **A**. Error-scaled difference distance matrices from pair-wise comparisons of all eight structures using ESCET program. The pdb codes and the bound inhibitors in the eight structures are: 1XNZ, **1**; 2EVM, **2**, 2EVC, **3**; 2Q92, **4**; 2Q93, **5**; 2Q94, **6**; 2Q95, **7**; and 2Q96, **8**. All changes in distances smaller than the threshold 1σ are shown in grey; differences between this lower limit and an upper limit of 8σ are shown using a colour gradient where red stands for expansion and blue for contraction, light colours represent small changes and dark colours large changes; all differences larger than the upper limit are shown as full blue and full red, respectively. The gradients used for colour coding are also shown separately at the bottom of the figure. **B**. Enlarged one of the matrices in A, showing the comparison between 1XNZ and 2Q96. For clarity, the matrices underwent 2 × 2 binning (maintaining the element with the highest absolute value in the respective binning area) before being displayed.

To further assess the differences among the eight complex structures, we performed pair-wise comparisons using Cruickshank's diffraction precision index [34] with the addition of linear B-factor scaling as implemented in the program ESCET [35,36]. The Cα carbons of residues 4–256 were used in calculating error-scaled difference distance matrices (Fig. 6). When the lower limit was set at the 4σ level (where σ is the uncertainty in the measurement of the difference), the only region that was found to be flexible was residues 61–67. If the limit was raised to 5σ, the flexible region narrowed to residues 62–66. The error-scaled difference distance matrices calculated by ESCET are consistent with the rmsd values presented in Table 2. This analysis confirms that the YHGY loop (residues 61–64) shows greater flexibility than other parts of the molecule in response to differing active site ligands.

### Comparison with the structures of human type I and type II MetAPs

**Figure 7:**
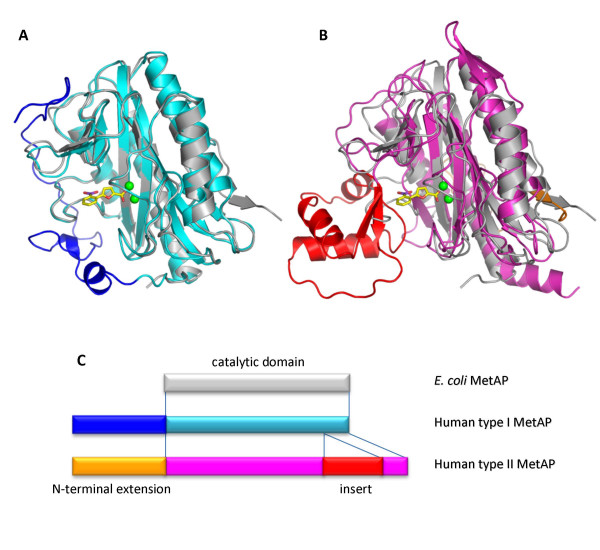
**Structural comparison between *E. coli *MetAP and human MetAPs**. Structure of *E. coli *MetAP used in the overlays is the one complexed with **4 **and coloured grey. (A) Overlay with human type I MetAP (PDB code 2NQ7) that is coloured cyan for the catalytic domain and blue for the N-terminal extension. (B) Overlay with human type II MetAP (PDB code 1B59) that is coloured magenta for the catalytic domain, orange for the N-terminal extension and red for the insert. (C) Schematic drawing of the domains in *E. coli *MetAP and human MetAPs.

We observed previously that some inhibitors of *E. coli *MetAP inhibit only human type I MetAP that is truncated at the N-terminus but not the full-length enzyme with an intact N-terminus [37]. As structures of both type I and type II human MetAPs are available, we aligned the structure of *E. coli *MetAP with those of the two human subtypes. *E. coli *MetAP is a typical bacterial MetAP, containing only a catalytic domain (Fig. 7C). On the other hand, mammalian MetAPs, including both type I and type II human MetAPs, have an N-terminal extension. Type II human MetAP has an extra insert, dissecting the catalytic domain into two fragments.

By overlaying the structures of *E. coli *MetAP and human type I MetAP, we see that the N-terminal extension of the human enzyme wraps around the enzyme surface and covers the YHGY loop (Fig. 7A). The surface-exposed loop in *E. coli *MetAP now becomes mostly buried in human type I enzyme. This could greatly reduce the plasticity of the loop and make the inhibitor binding pocket much less tolerant to structural variations in inhibitor molecules. This change is consistent with our previous observation on inhibition of the truncated and full length human type I MetAPs [37], and the partially buried nature of the loop could make it less adaptable for the inhibitors of differing size.

In addition, an overlaying of the *E. coli *MetAP and human type II MetAP structures reveals that the YHGY loop is not present at all in human type II MetAP, and instead, its position is now occupied by an insert, unique to type II MetAP (Fig. 4B). The insert of approximately 65 residues forms a distinct globular domain and is an integral part of the active site pocket. The adaptability of its binding pocket may be analyzed when more complex structures are available. However, differences in adaptability are likely found because the YHGY loop is part of the pocket in *E. coli *MetAP and it is substituted by the globular insert in human type II MetAP. It is interesting to note that the N-terminal extension of human type II enzyme is located away from the active site.

### Implications of the adaptability of the YHGY loop in developing MetAP inhibitors as antibiotics

Structure-based drug design takes advantage of the structural information of a medicinally important protein, especially at the active site, to guide the design and development of protein ligands to achieve desired potency and selectivity. Bacterial MetAP enzymes are the simplest in the MetAP family and contain only a catalytic domain. Most bacterial MetAPs, with exception of achaeal enzymes, belong to the type I MetAP family and are homologs. *E. coli *MetAP, as a typical bacterial MetAP, has high sequence homology with human type I MetAP within the catalytic domain (121 out of 264 residues are identical). It is certainly desirable to identify the differences between bacterial MetAPs and human counterparts so that MetAP inhibitors as potential antibiotics will selectively inhibit only bacterial MetAPs. The N-terminal extension and the insert in human MetAPs are the extra structural elements that can be potentially exploited to design selective inhibitors for bacterial MetAPs.

As more X-ray structures of *E. coli *MetAP in complex with inhibitors have become available, we now can compare the structures and characterize the binding of different inhibitors. Careful structural analysis of these structures reveals the adaptability of the YHGY loop (Y62, H63, G64 and Y65) that accommodates inhibitors of differing size. The adaptability of this loop in bacterial enzymes could be an important structural feature to exploit because the loop is partially occluded by the N-terminal extension in human type I MetAP [38-42] and is replaced by an unique insert in human type II MetAP [15]. Consequently, the ability to adapt multiple conformations within this loop of *E. coli *MetAP, and likely of other bacterial MetAPs, may not exist in human MetAPs and could be utilized to steer MetAP inhibitors towards selective inhibition of the bacterial enzymes.

This observation emphasizes the importance in considering the dynamics of ligand binding to enzymes in modeling inhibitors into a binding site on a protein, especially during the virtual screening of MetAP inhibitors. A rigid active site would appear to be relatively intolerant of anything but a nearly perfect fit or a slightly undersized ligand, but a flexible site would be more forgiving and tolerant of a wider range of structures. Molecules that might appear to fit poorly based on a rigid structure model may in reality fit quite well because of loop movement. Recognizing and utilizing this flexibility could be beneficial, for example, for optimizing the potency and selectivity of an inhibitor or fine-tuning its biopharmaceutical properties.

## Conclusion

Structural analysis of the complexes of *E. coli *MetAP with a series of related inhibitors reveals the ability of the surface-exposed loop containing the sequence YHGY to adapt multiple conformations to better complement the structural features of bound ligands. This adaptable loop likely exists in all bacterial MetAPs based on sequence similarity and the surface-exposed nature of the loop. However, this loop is partially buried by an N-terminal extension in the human type I MetAP and substituted by a globular insert in the human type II MetAP. The difference in ability of the substrate/inhibitor binding pocket to adapt to a wide range of ligand sizes may distinguish bacterial MetAPs from human MetAPs and could be exploited to design selective inhibitors of bacterial MetAPs.

## Methods

### Preparation of the protein and compounds

The recombinant *E. coli *MetAP was purified as an apoenzyme [30]. Compounds **1**–**3**, **6 **and **7 **were purchased from ChemBridge (San Diego, CA) and characterized by ^1^H and ^13^C NMR and high resolution mass spectrometry. Compounds **4**, **5 **and **8 **were synthesized in our laboratory. Their inhibitory activities on the Co(II)-, Mn(II)-, Ni(II)- and Fe(II)-forms of *E. coli *MetAP have been described previously [17,18].

### Crystallization Conditions

Initial crystallization conditions were determined using Crystal Screen and Index HT kits in 96-well sitting-drop plates (Hampton Research) at room temperature. Final crystals of the enzyme-inhibitor complexes were obtained independently by the hanging-drop vapour-diffusion method at 18–20°C. Inhibitors (200 mM in DMSO) were added to concentrated apoenzyme (12 mg/ml, 0.4 mM) in 10 mM MOPS pH 7.0. Hanging drops contained 3 μl protein solution mixed with 3 μl reservoir solution. The reservoir solution consisted of 10–15% PEG 20,000, 0.1 M MES (pH 6.5) and 0.2 mM MnCl_2_. The concentration ratio of inhibitor:apoenzyme was 5:1 for **4 **and **5 **and 10:1 for **6**–**8**, and that of metal:apoenzyme was 5:1.

### Data collection and structural refinement

Data were collected on an R-Axis IV imaging plate detector with a Rigaku rotating anode generator operated at 50 kV and 100 mA. Images were recorded over 180° in 0.5° increments at 100 K. Raw reflection data were indexed and integrated using MOSFLM [43] and merged and scaled using SCALA in CCP4 [44] with CCP4i interface [45]. Analysis of the estimated solvent content of each crystal [46] indicated only one molecule of the enzyme per asymmetric unit in all cases. The coordinates of our previously solved structure of *E. coli *MetAP (PDB code 1XNZ) with ligand, metal ions and water molecules removed were used as the search model for molecular replacement using MOLREP [47]. Crystallographic refinement was performed with CNS [48]. The refinement was monitored using 10% of the reflections set aside for free R factor analysis throughout the whole refinement process. Initial refinement started with simulated annealing with a starting temperature at 4000 K and 25 K drop in temperature per cycle. The models were refined with iterative cycles of individual B factor refinement, positional refinement, and manual model building using WinCoot [49]. The Mn(II) atoms were not included in the initial refinement procedure to reduce the model bias in phases and were then added to the model to the center of the peak in the Mn(II)-omitted *F*_obs_-*F*_calc _electron density map. The ligand and water molecules were added when the electron densities shown in 2*F*_obs_-*F*_calc _and *F*_obs_-*F*_calc _maps for their placement were unequivocal. The final 2*F*_obs_-*F*_calc _maps showed clear electron density for most of the atoms except for a few side chains at the molecular surface. The final models for all of the structures were analyzed using the program PROCHECK [50], and all have 99.6% of residues were in the allowed region of their respective Ramachandran plots. The atomic coordinates and structure factors for the structures have been deposited in the Protein Data Bank. Statistic parameters in data collection and structural refinement are shown in Table 1.

### Structural analysis

Structures were aligned with PYMOL [51] using the "align" command, and rmsd values were calculated with "rms" command after pair-wise alignment. The program ESCET [35,36] was used to make an objective analysis of the conformational variability of the eight structures 1XNZ, 2EVM, 2EVC, 2Q92, 2Q93, 2Q94, 2Q95 and 2Q96. All drawings for protein structures in the figures were generated using PYMOL.

## Authors' contributions

QQH and FJN prepared the ligands **4**, **5 **and **8 **used in this study. SXX carried out the crystallization experiments and data collection. ZQM performed the data processing and structural modeling. TDH carried out the computational ESCET analysis of the structures. QZY designed the study, carried out the structural analysis, and wrote the manuscript. All authors read and approved the final manuscript.
